# Understanding Pain Catastrophizing: Putting Pieces Together

**DOI:** 10.3389/fpsyg.2020.603420

**Published:** 2020-12-16

**Authors:** Laura Petrini, Lars Arendt-Nielsen

**Affiliations:** Center for Neuroplasticity and Pain, SMI, Department of Health Science and Technology, Faculty of Medicine, Aalborg University, Aalborg, Denmark

**Keywords:** catastrophizing, worry, catastrophic worry, rumination, behavioral inhibition and behavioral activation systems (BIS and BAS), interoceptive sensitivity

## Abstract

The present narrative review addresses issues concerning the defining criteria and conceptual underpinnings of pain catastrophizing. To date, the concept of pain catastrophizing has been extensively used in many clinical and experimental contexts and it is considered as one of the most important psychological correlate of pain chronicity and disability. Although its extensive use, we are still facing important problems related to its defining criteria and conceptual understanding. At present, there is no general theoretical agreement of what catastrophizing really is. The lack of a consensus on its definition and conceptual issues has important consequences on the choice of the pain management approaches, defining and identifying problems, and promoting novel research. Clinical and research work in absence of a common theoretical ground is often trivial. It is very surprising that clinical and experimental work has grown extensively in the past years, without a common ground in the form of a clear definition of pain catastrophizing and overview of its conceptual basis. Improving the efficacy and efficiency of pan catastrophizing related treatments requires an understanding of the theoretical construct. So far, most interventions have only demonstrated modest effects in reducing pain catastrophizing. Therefore, clarifying the construct may be an important precursor for developing more targeted and effective interventions, thereby easing some of the burden related to this aspect of pain. In our review, we have extracted and de-constructed common elements that emerge from different theoretical models with the aim to understand the concept of catastrophizing, which components can be modulated by psychological interventions, and the general role in pain processing. The analysis of the literature has indicated essential key elements to explain pain catastrophizing: emotional regulation, catastrophic worry (as repetitive negative thinking), rumination, behavioral inhibition and behavioral activation (BIS/BAS) systems, and interoceptive sensitivity. The present paper attempts to integrate these key elements with the aim to re-compose and unify the concept within a modern biopsychosocial interpretation of catastrophizing.

If you are distressed by anything external, the pain is not due to the thing itself, but to your estimate of it; and this you have the power to revoke at any moment

Marcus Aurelius, Meditations (T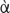 ε
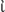
ς 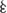
αυτóν)

## Introduction

In the past 30 years, *pain catastrophizing* generated substantial attention and was investigated in many clinical and experimental studies as an important correlate of pain, pain-related disability, and outcome (e.g., after surgery and chronification). Notably, in these years, more than 3,000 articles focusing on *pain catastrophizing* were listed in PubMed or Web of Science. This rising number of publications focusing on a psychological determinant in pain and pain chronicity emerged as a consequence of the embracement of the biopsychosocial model in western medicine at the end of the 20th century. In the same period, an important shift in paradigms was also observed in psychology with the introduction of cognitivism in the 1960s, leading to the emergence of the biopsychosocial perspective. It was in this framework that Ellis (1962) introduced the construct of *catastrophizing* for the first time.

In 1977, George Engel proposed the biopsychosocial model (Engel, 1977) as an opposition to the dominant biomedical model derived from Louis Pasteur’s germ theory of disease (1822–1895). The biomedical model provided important and fundamental advantages in medicine, but it was based on a dualistic mind-body viewpoint, and had its own basis in the reductionistic philosophy that dominated the field of medicine since the Renaissance (Gatchel et al., 2007).

The biopsychosocial approach has been particularly influential in the study of pain, since it expanded our knowledge of pain beyond an exclusive focus on its biomedical pathophysiology. The biopsychosocial model is now accepted as the most comprehensive perspective for the understanding and interdisciplinary treatment of chronic pain disorders (Gatchel, 2004; Gatchel et al., 2007), and cognitive variables such as pain catastrophizing have been recognized to modulate pain-related outcomes.

Despite this, the role of psychological interventions as therapy to reduce pain catastrophizing and their integrated roles in a biopsychosocial management framework are not fully developed.

In order to develop efficient interventions that achieve the best treatment outcome, it is imperative to understand the mechanisms and the potential factors that are behind therapeutic procedures (Day et al., 2012). For example, is the reduction in pain catastrophizing a therapeutic mechanism specific to cognitive-behavioral treatment for chronic pain? (Burns et al., 2012). Alternatively, are there other mechanisms and factors that can influence and interact with pain catastrophizing? Most importantly, what is pain catastrophizing when dissected into its different components, and which of these components can best be modulated by cognitive interventions?

Today, we acknowledge that pain catastrophizing is a multifaced complex construct but, unfortunately, its understanding does not expand beyond its pragmatic separation in rumination, magnification, and helplessness.

Pain catastrophizing is a major topic of discussion within the field of pain management, and a better understanding may eventually improve and validate cognitive intervention strategies. A variety of psychological approaches are used for the management of chronic pain. These span from classical cognitive-behavioral therapy (CBT) and or CBT-multimodal interventions to the more recent acceptance and commitment therapy (ACT) and mindfulness-based strategies (Burns et al., 2012). Although many of these are showing promising treatment outcomes, we are still lacking knowledge about the mechanisms underlying favorable effects of psychological therapies for chronic pain (Day et al., 2012).

Reduction in pain catastrophizing serves as index of changes in maladaptive cognitions, and therefore represents a therapeutic mechanism that works to reduce pain and improve functioning (Burns et al., 2012). However, when the intervention literature is analyzed, the effect size on pain catastrophizing is merely modest and unspecific (Schütze et al., 2018). This means that the reduction in catastrophizing scores associated with pain modulations are likewise observed in other therapeutic interventions that are not designed to or do not operate within a theoretical framework specific for targeting cognitive changes. Examples of such interventions are education interventions, physical exercise, or multimodal interventions (Burns et al., 2012; Schütze et al., 2018). Thus, the great variability observed in treatment options make it difficult to understand what mechanisms and variables of pain catastrophizing are subjected to change and can be underpinned as a potential target for helping individuals at catastrophizing less.

In order to improve the treatment efficacy of pain catastrophizing, this must be understood within a common theoretical framework.

Although several conceptualizations have been proposed, at the present time there is no general theoretical agreement of what catastrophizing really is and why it occurs. To date research on pain catastrophizing has grown enormously despite the absence of a common contextual framework.

Two extensive and thorough reviews provided by Sullivan et al. (2001b) and Quartana et al. (2009) have previously addressed the existence of multiple conceptual models of pain catastrophizing and noted the persistent controversies regarding catastrophizing and pain.

In the past decade, continuous investigations on pain catastrophizing have been conducted, adding novel elements that can help clarifying the construct. These investigations include emotional regulation and catastrophic worry as well as neuropsychological models of personality traits, the behavioral inhibition system (BIS), and the behavioral activation system (BAS). Expanding our knowledge and integrating these lines of research may improve our understanding of the underlying components of pain catastrophizing.

Thus, the aims of this narrative review are threefold: (1) de-composing early and modern conceptualizations of catastrophizing in order to highlight mechanisms and key-features underlying its concept; (2) re-composing catastrophizing by integrating these mechanisms and key-features within a biopsychosocial framework to generate a cohesive understanding of catastrophizing; (3) enhancing conceptual awareness on which psychological processes may play an important role in an interdisciplinary pain management framework.

Based on the available data, an integrated model is suggested for the mechanisms involved in pain catastrophizing to offer new possibilities to understand the nature of this construct, with the aim to improve multidisciplinary management of chronic pain.

## Methods

The present narrative review is intended as a comprehensive theoretical resource for addressing the above-mentioned aims. To our knowledge, no other study has tried to de-construct and recompose the concept of catastrophizing by extracting basic principles and processes from existing theoretical models. To our view, this step is fundamental in order to reach a comprehensive understanding of this psychological phenomenon and to improve pain management.

Studies cited in this review have been obtained from searches in PubMed, Web of Science, and PsycINFO databases, and through the authors’ familiarity with the published literature in the field. Clinical, observational and intervention studies, experimental, qualitative, and review articles were all included in the search. Articles have been identified using the following key-search: catastrophizing and pain catastrophizing and their relationship with rumination, worry, BIS and BAS, catastrophic worry and interoceptive sensitivity. Furthermore, a hand search of the relevant references has been undertaken to capture articles that might have been missed on the initial search. In addition, references in clinical psychology and pain psychology books have been consulted in order to have a broad overview of the available literature. The literature search has been performed in the period of March–April 2019 and has been updated again in March 2020.

Although this review follows to a large extent the guidelines for conducting systematic reviews, this is not a systematic review since the scope of this work is to provide a context for describing, elaborating, and evaluating a new conceptualization of pain catastrophizing based on the integration of the reviewed studies.

Studies have been included if they were peer-reviewed articles and published in English. References have been excluded if they did not address specifically the role of catastrophizing in relation to its theoretical framework or included it as one the primary variables.

The review is organized into four parts: (1) the importance of undertaking further steps in understanding the theoretical meaning of catastrophizing, (2) de-constructing the main identified key-features around the concept of catastrophizing starting from early approaches to more recent conceptualizations, (3) re-composition of all the key-features into one coherent view, and (4) the implications of an integrative view of catastrophizing at a theoretical, research, and clinical management level.

## Setting the Scene

Within pain literature, *catastrophizing* emerges as one of the most robust and reliable predictor and correlate of adverse pain experience (Sullivan et al., 2001a; Edwards et al., 2006; Weissman-Fogel et al., 2008; Traxler et al., 2019). For example, high levels of catastrophizing are associated with heightened pain intensity, increased pain severity, and emotional distress (Sullivan et al., 2001a; Turk and Okifuji, 2002; Keefe et al., 2004).

Studies have shown that catastrophizing is related to higher levels of pain and suffering (Lackner and Quigley, 2005), increased need for medical advice, greater health-care utilization (de Boer et al., 2012), increased disability (Picavet et al., 2002), and worse outcome after surgery (Hovik et al., 2016). Remarkably, pain catastrophizing is also associated with pain outcomes in experimental pain studies in a pain-free population (Edwards et al., 2004; Campbell et al., 2010; Kristiansen et al., 2014).

Although catastrophizing is viewed as a key element for understanding variability in pain response, there are many important questions to be answered about what catastrophizing really is and why it occurs. For example, *why are some people trapped in catastrophic thinking, which is difficult to disengage from?*

Despite its increasing use in both clinical and experimental studies, the concept of pain catastrophizing has often generated silent concerns in relation to its definition and conceptualization. Particularly important is to understand the nature and function of the phenomenon that we refer to as *pain catastrophizing*.

There are several issues and shortcomings associated with the concept of catastrophizing that result in a circular problem.

In experimental and clinical pain research, there is a tendency to define pain catastrophizing merely and mainly as the content of its measuring scales such as the catastrophizing scale of the Coping Strategy Questionnaire (CSQ) (Rosenstiel and Keefe, 1983) or the Pain Catastrophizing Scale (PCS) (Sullivan et al., 1995). Unfortunately, this tendency has been demonstrated to be flawed.

As questioned earlier by Turner and Aaron (2001), and recently addressed by Crombez et al. (2020) none of the pain catastrophizing scales can specifically measure pain catastrophizing. Instead, the content of the pain catastrophizing measures is better explained by pain-related worrying or pain-related distress (Crombez et al., 2020).

These findings (Crombez et al., 2020) support the doubts raised about whether pain catastrophizing measures assess pain catastrophizing as defined in the cognitive-behavioral literature (Turner and Aaron, 2001; Eccleston et al., 2012; Eccleston and Crombez, 2017). In fact, many authors consider pain catastrophizing as an extreme instance of worrying. This has been previously suggested by psychological theories of anxiety disorders (Davey and Levy, 1998). Within the anxiety literature, the tendency to catastrophize has been identified as exacerbating the adverse effects of pathological worry (Kendall and Ingram, 1987; Kendall and Hollon, 1989). Catastrophizing involves “dwelling on the worst possible outcomes of any situation in which there is a possibility of an unpleasant outcome” (Beck and Emery, 1985).

Turner and Aaron (2001) emphasized the importance to integrate the advancement in the field of anxiety and worry into pain research in order to better understand the nature and the function of pain catastrophizing.

Flink et al. (2013) published a stimulating paper that integrated catastrophic worry into a novel conceptualization of pain catastrophizing. The concept of catastrophic worry was inspired by the literature on anxiety disorders (Borkovec et al., 1983; Davey and Levy, 1998) and combined with new advances in pain research. This new concept considered catastrophizing as a part of the worry process (Eccleston and Crombez, 2007) highlighting the similarities between different forms of repetitive negative thinking. This is driving a process of clarification even though experimental studies testing this model are still lacking.

If there are problems with the measuring instruments and the definition of pain catastrophizing, then it is not surprisingly that we observe incongruencies in treatments outcomes.

## What Is Catastrophizing?

The first question that needs a unified answer is *what is catastrophizing*?

The word has a strong connotation, is emphatic and tends to magnify the meaning of the content. The word induces a strong visual image especially if we turn our mind to a natural disaster. One interpretation of a catastrophe used within emergency services, is a disaster where the resources available exceed what is required for its mitigation. That is perhaps an apt analogy to catastrophizing pain patients where the pain exceeds the mental capability of the patient to cope with the pain.

In the scientific literature, catastrophizing is generally seen as a negative cognitive process. In the past, the construct of catastrophizing has grown-up mostly in parallel within two lines of research: (a) psychological research on anxiety and depressive disorders, and (b) pain research. In recent years, psychological research has also connected catastrophizing to several psychological disturbances such as phobias, obsessive-compulsive disorders, eating disorders, and many others.

In response to stress and anxiety some individuals focus on the negative aspects of a situation and expect the worst outcome (Ellis, 1962). This process is frequently referred with many terminologies: cognitive errors, dysfunctional cognitions, negative-self statement ideation, catastrophizing or simply negative thinking (Beck, 1976; Beck et al., 2005). These cognitive aspects have been studied for many years in an attempt to understand anxiety-related conditions and depression (Beck, 1976; Kendall and Ingram, 1987; Beck et al., 2005).

Likewise, similar cognitive processes have been identified in response to laboratory and clinical pain conditions and have been represented under the term *pain catastrophizing*, which is often broadly conceived as an exaggerated mental set brought to bear during painful experiences (Sullivan et al., 2001b) without narrowing it down to the actual components. In addition, very little is known about the differential effects of the specific components of catastrophizing on pain-related outcomes (Craner et al., 2016).

At present, the construct of pain catastrophizing has some inbuilt theoretical problems that call for clarification. Although pain catastrophizing is viewed as vital for understanding variability in pain responses, there is no common theoretical view on what pain catastrophizing really is and why it occurs. Several competing theoretical frameworks have been suggested. A schema-activation model appears to be a useful theoretical framework by which to conceptualize the catastrophizing-pain relationship, but unfortunately there is a debate about whether catastrophizing should be viewed as a cognitive schemata, a coping strategy or a personality trait (Gilliam et al., 2010). Alternatively, pain catastrophizing has also been considered as a communal coping response by which an individual utilizes exaggerated pain expressions as a way of eliciting assistance or empathic responses from others (Sullivan et al., 2001b).

Recently, pain catastrophizing has been reframed as an unsuccessful problem-solving strategy (Eccleston and Crombez, 2007), or as a catastrophic worry (Flink et al., 2013). Catastrophic worry is interpreted as a repetitive negative thinking strategy to regulate negative emotional responses.

Despite all these models, the theoretical underpinning of pain catastrophizing has not been extensively researched, instead empirical research has merely used the construct of pain catastrophizing as it has been framed by the PCS (Sullivan et al., 2001b). Although several other assessment tools exist, such as the *Cognitive Error Questionnaire* (CEQ) (Lefebvre, 1981), the *Coping Strategies Questionnaire* (CSQ) (Rosenstiel and Keefe, 1983), *Cognitive Coping Strategies Inventory* (CCSI) (Butler et al., 1989), *Pain Cognition List* (PCL) (Vlaeyen et al., 1990), and *Pain-Related Self-Statements Scale* (PRSS) (Flor et al., 1993).

The PCS taps three dimensions of catastrophizing, which are related with the predisposition to ruminate, magnify, and feel helpless about pain (Sullivan et al., 1995). In this context, catastrophizing is defined as *an exaggerated negative mental set brought to bear during actual or anticipated painful experience* (Sullivan et al., 2001b).

Broadly, pain catastrophizing is characterized by the tendency to magnify the threat value of the pain stimulus and to feel helpless in the context of pain, and by a relative inability to inhibit pain-related thoughts in anticipation of, during or following a painful event (Quartana et al., 2009).

In recent years, sporadic efforts have been made to unify these different lines of research. Catastrophizing has been suggested as a transdiagnostic construct identified across different disorders (Linton, 2013; Gellatly and Beck, 2016).

## Decomposing Catastrophizing

This section analyzes and decomposes psychological and pain-related catastrophizing conceptualizations from early to recent approaches in order to identify key-features and mechanisms.

### Early Psychological Approaches to Catastrophizing

#### Albert Ellis; Irrational Beliefs

Ellis (1962) coined the term *catastrophizing* within the psychological framework of REBT (Rational Emotive Behavior Therapy) as a factor that emerges in some forms of psychopathology such as anxiety and depression. Ellis proposed that both rational and irrational beliefs guide human behaviors, goals, and actions, and in turn they affect emotional responses. Ellis hypothesized that catastrophizing stemmed from an underlying core of irrational belief. Irrational beliefs tend to be an obstacle for achieving personal goals. For example, having thoughts like *this is awful*; *it is the end of the world*, or *this treatment will ruin my life!* will induce negative emotional responses and prevent proper behaviors.

Here, catastrophizing is seen as a prediction of a negative outcome, and as a forecast for the worst conclusion. Catastrophizing is the primary element of emotional dysfunction since in this view irrational ideas are assumed to be causing psychological distress.

#### Aaron Beck; Cognitive Schemata and Automatic Thoughts

After Ellis’s first observations, Beck (1976) (the father of cognitive therapy, which is one the therapeutic approaches within the larger group of cognitive behavioral therapy, CBT) used the construct of *catastrophizing* within his cognitive theoretical models of depression and anxiety to describe maladaptive cognitive styles observed in patients affected by these disorders. Beck (1976) and Beck et al. (2005) developed a model of cognitive distortions where a central role was played by the activation of automatic thoughts. According to this model, *catastrophizing* is considered an automatic cognitive error. Catastrophizing was defined as the tendency to anticipate danger, and to perceive a total disaster (e.g., *I won’t be able to function at all*) as the most probable outcome without considering other more likely consequences (Beck et al., 2005).

In this view, some individuals automatically engage in catastrophic cognitions when they are facing threatening situations. Threatening situations trigger overemphasizing the probability of a catastrophic outcome and exaggerate the possible negative consequences of its occurrence (Beck, 1976; Beck et al., 2005).

#### Other Psychological Approaches: The Role of “Worry”

Psychologically, it is the perception of a threat that initiates the anxiety response. Detecting a threat creates a problem to solve, mainly in those situations where avoidance behaviors are not possible. Thus, cognitive activity is the only tool available. Cognitively, some individuals respond to a perceived threat by *worrying* (Ruscio et al., 2001). Therefore, worry is the cognitive attempt to solve the problem of a possible future danger. Individuals use worry to avoid or to prepare themselves to cope with future threat (Borkovec et al., 1983). Indeed, anxious individuals believe that viewing events in the most negative possible way is beneficial. For example, *it cannot get any worse than this, so if I am prepared for it, then I will be prepared for anything* (Borkovec et al., 1983). In this way, thoughts are biased toward the most negative and catastrophic outcomes. In the psychological literature, worry has been defined as *a chain of thoughts and images negatively affect-laden and relatively uncontrollable* (Borkovec et al., 1983), whereas catastrophizing has been identified as *the process that exacerbates the adverse effects of pathological worry* (Kendall and Ingram, 1987). Thus, excessive worry occurs through the process of catastrophizing, in which individuals persistently re-iterate the problematic features of their worry topic. Catastrophizing involves the worrier to persistently posing internal automatic questions: *what if …?* (Kendall and Ingram, 1987).

The process of catastrophizing is *per se* not an effective method since instead of bringing the worry problem to a solution it leads to a progressively worse scenario (Hazlett-Stevens and Craske, 2003). Davey and Levy (1998) identified the catastrophic process with the term: *catastrophic worry*, which reflects personal inadequacy and perseverative iterative style due to unsuccessful attempts in problem solving. Vasey and Borkovec (1992) found that chronic worriers generated more imagined catastrophes than non-worriers, and that the process of catastrophizing was associated with an increased negative affect.

#### Brief Summary

From these earlier psychological interpretations, catastrophizing emerges as a key cognitive factor in emotional dysregulation. Maladaptive cognitions (beliefs or schemas) contribute to the maintenance of emotional distress and behavioral problems, and give rise to specific and automatic thoughts, such as catastrophizing. Especially, in situations where threat is perceived or detected. Worry is related with catastrophizing as “an automatic questioning style” (a “*what if … happens*” style of thinking) that leads to a further distortion of the appraisal of the threat.

### Early Pain Research: Interpreting Catastrophizing in Pain

In early pain research, there was an interest in investigating factors responsible for variability in pain responses among individuals subjected to an identical nociceptive stimulus (Neblett, 2017). A behavioral dichotomy was consistently observed based on the ability to tolerate pain. That is, individuals that can tolerate pain for a short period of time as compared with individuals that can tolerate pain for a long period of time (Spanos and Brazil, 1984; Chen et al., 1989). When examined, the two groups differed primarily in the type of cognitions they reported during a pain experience (Chen et al., 1989). Negative cognitions were observed in the most sensitive group. Negative cognitions were identified by classifying participants in *catastrophizers* or *non-catastrophizers* (Spanos et al., 1979; Chaves and Brown, 1987). Catastrophizers were individuals who reported worry, fearful, anxious thoughts, focused on, and exaggerated the unpleasantness of the situation, and who were unable to shift attention away from the pain. In line with these investigations, *catastrophizers* had an enhanced pain perception associated with worry and anxiety about the pain, imaginative negative consequences, and thoughts about more severe situations.

#### Brief Summary

In these early qualitative pain studies, catastrophizing interpretations were inspired by the concepts of anxiety and worry within the field of psychology. Catastrophizing was associated with elements of pain related worry, fear, magnification, and inability to cope with pain.

### Recent Pain Research: Pain Catastrophizing – From Fear to Worry?

After these initial observations, there was a shift in pain research into looking for important psychological determinants of chronic pain adjustment. Catastrophizing was found to be an important pain predictor, which could partially explain the relationship between pain severity and adjustment. In the pain literature, catastrophizing refers to a broader type of dysfunctional thinking toward pain. This includes difficulty in shifting attention away from pain, perceiving pain as unusually more intense, and feeling helplessness in controlling pain (Sullivan et al., 1995). This literature suggests that although depressive cognitive errors and pain catastrophizing share some commonalities, catastrophizing is a separate construct since it predicts pain outcome even when depression is statistically controlled (Sullivan and Neish, 1998; Geisser et al., 1999; Keefe et al., 1999). Several theoretical constructs have been proposed for pain catastrophizing, and initiated a debate on whether it should have been considered a coping strategy (Keefe et al., 1999), an appraisal model (Haythornthwaite and Heinberg, 1999; Thorn et al., 1999), a maladaptive pain belief (Geisser et al., 1999), a cognitive error on the base of Beck’s view (Jensen et al., 1991), or a communal copying strategy. Likewise, the importance to distinguish process from outcome (Geisser et al., 1999) was also part of this debate. Unfortunately, these discussions never concluded in a definitive conceptual agreement on catastrophizing but instead underlined the controversy over the meaning of this construct (Geisser et al., 1999).

Table 1 shows an overview of the different theoretical interpretations.

**TABLE 1:** An overview of the different theoretical conceptualizations of catastrophizing.

Conceptualization	Definition	Source
Cognitive schemata and negative thoughts	Catastrophizing is seen a part of the pattern of thoughts (schemata) that guide cognitive processes and behaviors. Catastrophizing is seen as a negative or unwanted thought.	Beck and Emery (1985)
Coping strategy	Catastrophizing is seen as a cognitive effort to manage specific external and/or internal demands brought on by pain experiences that are appraised as burdensome.	Keefe et al. (1999)
Maladaptive pain belief	Catastrophizing is defined as cognition (thoughts) on pain problems. Maladaptive beliefs about pain – for example that pain is a signal of damage or harm to the body or that pain is disabling.	Geisser et al. (1999)
Secondary appraisal	Catastrophizing is seen as the evaluation of the ability to cope with the situation – credence on coping capabilities.	Thorn et al. (1999), Lazarus and Folkman (1984).
Personality trait or situational state	Catastrophizing is seen as stable characteristic of the individual (personality-based) that is present across different situations. Catastrophizing is seen as a response that varies over time and is determined by situational factors.	Sullivan et al. (2001b)
Communal coping strategy	Catastrophizing is seen as a way to elicit and/or maximize social support.	Sullivan et al. (2001b)
Misdirected problem-solving	Catastrophizing is seen as part of the worry process that has the function to actively solve a problem. In this context, catastrophizing is seen as an unsuccessful problem-solving strategy.	Eccleston and Crombez (2007)
Catastrophic worry process	Catastrophizing is seen as a repetitive negative thinking which is abstract, intrusive, and difficult to disengage. It is seen as a mediator of emotional regulation.	Flink et al. (2013) and Linton (2013)
BIS–BAS model of pain	Catastrophizing is seen as BIS-related cognition, within individual personality traits characteristics.	Jensen et al. (2016)

Meantime a variety of non-specific short-term cognitive treatments have been shown to be effective in reducing catastrophizing by pain patients. Although not specifically intended to reduce catastrophizing, certain cognitive therapy techniques that instructed patients to de-catastrophize, were effective in a cognitive-behavioral approach for pain management (James et al., 1993; Thorn et al., 2002, 2007).

At present, there is still a need for optimization of psychological therapy for reducing pain-related catastrophizing thinking. A recent review with meta-analyses (Schütze et al., 2018) showed that different types of intervention techniques are capable to reduce pain catastrophizing, nevertheless the estimate of these changes indicated only a modest effect. Surprisingly, the results showed that not only interventions designed to target pain catastrophizing (such as cognitive restructuring in CBT) reduced pain catastrophizing but also other types of treatment approach, such as physical exercise or combination of CBT and physical exercise. In addition, these findings suggested that high levels of heterogeneity among interventions, missing information regarding the manualization included in the treatment, and the lack of a consensus about the theoretical construct of pain catastrophizing make difficult to evince conclusive results.

In parallel to this debate, a new model, within the biopsychosocial framework, the fear-avoidance model (Vlaeyen and Linton, 2000; Vlaeyen et al., 2016) was developed. This model provided a description of the path followed by individuals experiencing acute pain, who may, subsequently, become trapped into a vicious cycle of chronicity and suffering.

In the fear-avoidance model, catastrophizing was conceptualized as a cognitive key element for generating fear and avoidance behaviors. Indeed, catastrophizing was identified as the turning point at which individuals either enter or not enter the fear-avoidance cycle. The cycle is initiated if pain is misinterpreted as a catastrophe. Catastrophic misinterpretations of pain lead to an extreme fear of experiencing more pain or having a (re)injury, which progressively extend to the fear of physical movements, and finally to a total avoidance of movement and activity. Long-term avoidance of physical activity has several consequences, from impairing functioning and by making an individual more physically weak, to increase negative mood that contributes to create a psychological feeling of disability, which if protracted can lead to depression. Consequently, the model explains how a susceptible individual enters in a dangerous loop of pain amplification and disability based on the tendency of an individual to catastrophize (Vlaeyen and Linton, 2000; Vlaeyen et al., 2016).

Although the fear-avoidance model has been successful and numerous studies have supported the role of catastrophizing in maintaining dysfunctional behaviors responsible of chronicity and disability (Keefe et al., 2000; Severeijns et al., 2001; Hirsh et al., 2011), continuing research questioned the sequential relationship proposed by the original model (Pincus et al., 2010; Slepian et al., 2020). Thus, pain catastrophizing might not be seen as an important driving factor in this model.

In addition, the model did not consider or explain why some individuals catastrophize, and whether they all express or experience fear (Asmundson and Katz, 2009). In relation to the latter, it has been argued that individuals might experience a future oriented threat, which is better described as an *anxiety emotional response* rather than a *fear emotional response* (Asmundson and Katz, 2009). Although the differences between fear and anxiety could be blurred and the terms used interchangeably; fear and anxiety are different. Both responses are necessary components of adaptive behavior, but whereas *fear* is characterized by a response to a *present-oriented-threat stimulus, anxiety* is a response to a *future-oriented-threat stimulus*. Therefore, it has been suggested that it would be more appropriate to describe these behaviors in terms of pain related anxiety (Asmundson and Katz, 2009).

Since it is the perception of threat that initiates the anxiety process, then it is reasonable to suggest that worry is one of the significant ways in which individuals respond to the perceived threat. As discussed previously, worry has the function to prioritize threat and promote problem solving. Therefore, catastrophizing should be considered within the worry context.

Often, people with pain perceive pain as a signal of aversive threat, and want to find a solution for their condition, a solution that unfortunately, cannot be found (Aldrich et al., 2000). Consequently, these patients perceive pain as an unsolved problem (Aldrich et al., 2000). It has been suggested that this experience is phenomenologically similar to worrying, where no immediate solution is available (Crombez et al., 2012). Taking this into consideration, new conceptualizations of pain catastrophizing have indicated *worry* as a central element to characterize catastrophizing. Aldrich et al. (2000) have re-framed the negative and threatening thinking about chronic pain within the context of worry. Chronic pain is re-represented as chronic vigilance to the threat of pain that leads to perseverative attempts at solving the problem of escaping from pain. The prolonged experience of inescapable pain generates high level of awareness about one’s body (Bacon et al., 1994), high difficulty to disengage from pain (Van Damme et al., 2004), and high levels of symptoms reporting (Ciccone et al., 1996). Furthermore, the repeated attempts to solve an insoluble problem provoke frustration and increase self-referent negative thinking that may become cognitively fixed or locked within self-perpetuating rumination (Aldrich et al., 2000).

Eccleston and Crombez (2007) proposed a new model of pain-related worry that was explained as a form of *misdirected problem solving*. In this model, worry motivates patients in engaging in possible solutions to resolve the pain, but when the solution attempts fail, worry is regenerated to establish new problem-solutions. The patient is therefore trapped in a *perseverative loop* of misdirected problem solving with no ending.

In addition, Flink et al. (2013) and Linton (2013) outlined the importance of including *worry* as part of the catastrophic process, by introducing the term *catastrophic worry* (Davey and Levy, 1998), to emphasize the notion that pain catastrophizing has an in-built relation with worry, as suggested in the misdirected problem-solving model (Eccleston and Crombez, 2007), together with the assumption that catastrophizing has a coping function, as suggested in the communal coping model (Sullivan et al., 2001b). In this new integrated view, catastrophic worry is proposed as a form of negative repetitive thinking, which has the purpose to reduce negative emotions triggered by pain or by other stimuli (exteroceptive or interoceptive).

However, as argued earlier, engaging in catastrophic worry is an infective strategy, since it does not produce any real solution to the problem, but only a *cognitive avoidance* of the threat through a perseverative thinking activity (catastrophic worry process). Individuals engaging in catastrophic worry hold beliefs that this activity will reduce negative emotions triggered by pain or other somatic stimuli. In this context, catastrophic worry is seen as a mediator of emotional regulation, a process aimed to reduce the effects of negative emotions.

Paradoxically, as it will be discussed later, engaging in catastrophic worry produces instead an opposite effect, which magnifies the emotional-pain related response leading to a reinforcement of the catastrophic worry cycle.

In our opinion, explaining pain catastrophizing as an emotional regulator has many advantages and can be a very useful framework to elucidate mechanisms of pain chronicity and disability. This will be discussed further in the next paragraphs.

#### Empirical Support in Pain Research

Studies are supporting the view that worry plays a central role in the cognitive and emotional framework of chronic pain patients. In an early study (Eccleston et al., 2001), patients with chronic pain reported that pain-related worries were intrusive, attentional demanding, difficult to stop, and distressing. The study also provided an analysis of the content of worry, which included both pain-related and non-pain related worries. The most common theme of pain-related worry was medical uncertainty (e.g., *is this a new pain? why has the intervention made it worse?*), followed by disability (e.g., *I can’t do the ironing*), pain experience (e.g., *this pain just keeps hurting*), and negative affect (e.g., *I am useless*).

The study broadly supports the view of Aldrich et al. (2000) for how a normal response to somatic threat exacerbates the suffering associated with chronic pain. The results show that worrying about threat continues to maintain vigilance on the object of threat (Mogg et al., 1990; Hirsh et al., 2011). In other words, worrying about chronic pain maintains the vigilance for pain, resulting in a persisting feeding of the perceptual threat.

Other empirical investigations are supporting the view that worry about chronic pain may promote awareness to an insoluble problem. In this perspective, chronic pain patients are not passive victims of anxiety, but are active participants engaged in a constant process of evaluating and interpreting threats, their possible consequences, and potential solutions (De Vlieger et al., 2006).

Likewise, a study from De Vlieger et al. (2006) supports the idea that worry is a common feature in chronic pain patients. Remarkably, worry does not seem to have a psychopathological component in these patients, as it has been found in individuals with generalized anxiety disorders. Chronic pain patients do not show deficits in general problem-solving appraisal or confidence. Instead, what emerges as atypical, is the extent to which patients attempt to engage in solving an insoluble problem. The idea that worry operates as an *experiential avoidance* for controlling emotional-related threatening information associated with pain is further supported by a study of Lackner and Quigley (2005). Their study showed that chronic pain patients with high levels of worry engage in more catastrophic thinking leading these patients to experience more pain and suffering.

### Recent Psychological Approaches: The Role of “Rumination”

Rumination and worry, although they share many features in common and can be experienced together, should be considered two separate and distinguishable forms of negative thinking (Fresco et al., 2002; Nolen-Hoeksema et al., 2008).

In general terms, rumination is a form of circular thinking that swallows the individual in a path without a way out, and it can be broadly defined as *perseverative self-focused thinking process*, whereby an individual goes over and over the same thoughts in his or her mind. This process generally interferes with a person’s ability to inhibit thoughts, generate alternative ways of thinking, and switch the focus of attention. Consequently, rumination is a process of perseverative thinking about one’s feelings and problems. Although, there is not yet a common theoretical view about rumination (Smith and Alloy, 2009), the most accredited model is the one proposed by Nolen-Hoeksema in the Response Style Theory (Nolen-Hoeksema, 1991). According to this model, rumination is a way of response to distress. Rumination involves repetitively and passively focusing on distress symptoms as well as on their possible causes and consequences.

Alike worry, rumination does not lead to an active problem solving. Individuals, who ruminate, remain fixated on their problems and feelings without taking actions (Nolen-Hoeksema et al., 2008). In this way, rumination interferes in engaging in effective problem solving and inhibits the initiation of positive behaviors, because it makes negative cognitions more accessible (Nolen-Hoeksema, 1991).

#### Empirical Support in Pain Research

At present, only few studies have investigated the direct link of rumination and chronic pain. Clinical observations suggest that patients with chronic pain spend a lot of time ruminating about their pain (Edwards et al., 2011).

Qualitative studies that have analyzed the content of ruminative thoughts have shown that chronic pain patients held a number of positive beliefs about rumination (e.g., helping in coping, problem-solving, and avoiding repeating mistakes) as well as negative beliefs (e.g., rumination is uncontrollable) (Edwards et al., 2011; Schütze et al., 2017).

Individuals, which are prone to ruminate, experience prolonged dysphoric reactions to problems, and they are more negatively biased in interpreting and solving problems (Lyubomirsky and Nolen-Hoeksema, 1993; Nolen-Hoeksema et al., 2008). In addition, the hypothesis that metacognitions (beliefs about their own thinking and their own coping strategy) may influence the selection of rumination is common in many theories [Response styles theory (RST), Stress-Reactive Rumination (S-REF), the Goal-Progress Model, Post-Event Processing] (Smith and Alloy, 2009).

Recent studies support the importance of the metacognitive aspect of *rumination* when characterizing pain catastrophizing (Schütze, 2016; Schütze et al., 2020). Many pain patients with high levels of catastrophizing believe that rumination help to solve problems and to prepare handling future threats, even when the sense of uncontrollability over ruminative thoughts produces negative consequences on mental health (Schütze et al., 2017).

In addition, quantitative studies using the PCS have shown that the different PCS sub-categories, such as rumination, magnification, and helplessness, might be better in explaining the associations between pain and disability when examined independently.

Rumination has been associated with symptoms magnification and poor clinical outcome (Sansone and Sansone, 2012), it has been seen as a major predictor in the severity of patients’ disability, and it has been strongly correlated with pain intensity ratings (Sullivan and Neish, 1998). In addition, cross-sectional studies have shown that rumination PCS sub-scale was able to differentiate between pain patients from healthy volunteers (Osman et al., 2000), and that rumination and helplessness sub-scales, but not magnification, were correlated with bodily pain (Nijs et al., 2008).

Although the PCS can help to provide evidence about the presence of rumination in pain patients, it does not provide any information about the causes or content of rumination. Recently, a pain metacognition questionnaire assessing beliefs underlying rumination about pain has been developed (Schütze et al., 2019). This additional tool can help future clinical and experimental research to clarify ruminative style in pain.

### Catastrophizing as an Emotional Regulator

A way to understand how catastrophizing impacts pain perception is to conceptualize it as part of the emotional regulator process. Pain is an emotional experience. Thus, understanding how individuals use various emotion regulation strategies in daily life, and how these strategies affect pain-related emotional experience on short- and long-term is an important factor.

Emotion regulation refers to a process by which individuals can influence the course and expression of their emotions (Gross, 1998, 2002), and it is essential for maintaining a healthy life. Conversely, emotion dysregulation characterizes mood, anxiety, and pain disorders (Breivik et al., 2014).

Gross (1998) distinguishes two important groups of strategies to regulate elicited emotions: (i) *re-appraisal*, characterized by modifying the meaning of an emotional stimulus; and (ii) *suppression*, characterized by inhibiting or reducing the emotion-expressive behavior elicited by a stimulus. Research has shown that reappraisal is more efficient than suppression in reducing negative emotional experience (Gross and Levenson, 1997).

It is argued that rumination and worry instantiate a superordinate process known as *repetitive negative thinking* (Fresco et al., 2002). Repetitive negative thinking is defined as *a style of thinking about one’s problems (current, past or future) or negative experience (past or anticipated) that is repetitive, at least partly intrusive, and is difficult to disengage from* (Ehring and Watkins, 2008; Ehring et al., 2011).

Repetitive negative thinking is considered a form of avoidant copying strategy (Stroebe et al., 2007; Flink et al., 2013), and individuals engage in this thinking to prevent confrontation with worse feelings and thoughts (Stroebe et al., 2007). Occupying one’s thoughts with repetitive negative thinking prevents confrontation with the threat. For example: *what I could have done to prevent this situation?* In addition, repetitive negative thinking can be negatively reinforced by abstract cognitive activity; for example: *Why do I suffer from pain?* This form of abstract thinking impedes activation and processing of emotional and somatic responses (Flink et al., 2013).

Flink et al. (2013) proposed to re-conceptualize pain catastrophizing as form of *repetitive negative thinking.* Pain patients engage in catastrophizing or repetitive negative thinking to reduce the aversive physiological and psychological aspects of the threatening pain-related situation. This conceptualization is very similar to the definition from psychological literature on worry and anxiety.

The authors labeled this form of thinking *catastrophic worry.* The introduction of the term *catastrophic worry* is to our view quite appropriate since from one side it incorporates this superordinate process of repetitive negative thinking; from the other side, it underlines the similarities with other forms of repetitive negative thinking, such as worry, rumination and catastrophizing, that we have observed in the literature. Furthermore, it reinforces the emerging transdiagnostic literature that wants to identify common processes among different disorders (Linton, 2013; Gellatly and Beck, 2016).

It has been hypothesized that patients engage in *catastrophic worry* in an attempt to downregulate negative emotions arising from a stressful situation such as persistent pain or distress (Flink et al., 2013; Linton, 2013).

Regulatory strategies employed by emotion and pain are surprisingly similar (Linton and Bergbom, 2011; Linton, 2013). Strategies for pain control also include distraction, re-appraisal, withdrawal/avoidance, self-reward, and suppression (Linton, 2013).

#### Empirical Support in Pain Research

Individuals with excessive worrying show deficits in emotion regulation. Specifically, they seem to not be able to accept negative emotions (Salters-Pedneault et al., 2006) and as a consequence they show an increase in negative affect and dysfunctional beliefs (Le Borgne et al., 2017).

Recent experimental studies support the hypothesis that an active emotional manipulation would directly influence the experience of pain. For example, Lefebvre et al. (2017) demonstrated that a manipulation of emotions had significant effects on the experience of worry about pain, and that a direct manipulation of pain-related worry increased pain intensity (Lefebvre and Jensen, 2019).

In addition, suppression was found to mediate the relationship between negative affect and pain catastrophizing (Wong and Fielding, 2013). Gilliam et al. (2010) showed that catastrophizers tend to suppress negative emotion and, as a consequence of this mechanism, experience prolonged recovery and long-term adjustment to pain.

Similar results were also reported in an experimental manipulation of suppression, where catastrophizers reported increased muscle tension at the site of injury (Quartana et al., 2007).

Although suppression might seem a positive adaptive strategy, since the goal is to keep the emotion and/or pain out of the mind, several studies have shown that this process often produces the opposite effect (Magee et al., 2012). Indeed, attempts to suppress unwanted thoughts, including those regarding pain and distress, may paradoxically increase awareness and salience of the thoughts and feelings that an individual wants to avoid (Wegner et al., 1987). Efforts to suppress pain-related thoughts either during or prior to pain had the unintended effect of increasing reports of pain severity in comparison with individuals who did not attempt to suppress pain (Cioffi and Holloway, 1993; Sullivan, 1997).

To our view, the process of suppression/avoidance could in fact magnify the negative emotions and consequently fuel the catastrophic worry cycle.

### Behavioral Inhibition and Activation Systems (BIS/BAS) in Emotion Regulation

If catastrophic worry is a form of emotional regulatory process, the question is then what factors contribute to the development and maintenance of catastrophizing or catastrophic worry? In simple words: *what factors make an individual more prone to catastrophize?*

Recently, Jensen et al. (2016, 2017) proposed the BIS–BAS model of pain where key psychological factors are used to predict pain responses and behaviors. The model is derived from Gray’s biological personality theory (*Reinforcement Sensitivity Theory, RST*), which postulates two main dimensions of personality: anxiety proneness and impulsivity (Gray, 1982, 1987). According to this theory, individual differences in personality and behavior can be explained by the activity of two distinct neurophysiological systems, the BIS and BAS that coordinate adaptive behaviors in response to relevant environmental cues. BAS regulates appetitive motivation whereas BIS regulates aversive motivation. The BIS–BAS system has been related with the neurobiological activity of the motivational and reward system in the brain.

Behavioral inhibition system is sensitive to signals of danger and punishment by detecting threatening possible stimuli or events and enhances *avoidance behavior* (*suppression*). *BAS*, instead, is sensitive to signals of reward, non-punishment, and escape from punishment by encouraging *approach behaviors* (*re-appraisal*). Since pain is usually perceived as a threatening and/or punishing stimulus, the BIS–BAS model of pain hypothesized that the BIS system is involved in pain (Jensen et al., 2016).

Several studies have shown that different types of BIS–BAS sensitivity are associated with specific clinical pathologies. For example, people with a sensitive BIS are prone to present problems of anxiety and depression (Maack et al., 2012; Hundt et al., 2013), and tend to worry and ruminate (Corr, 2004) due to excessive attention to cues related to negative events. BIS facilitates negative emotions and enables behavioral interruption and inhibition. BAS reactivity is associated with an orientation toward reward and impulsivity. BAS is also linked to positive affect (Meyer and Hofmann, 2005). However, people with highly sensitive BAS are particularly vulnerable to the development of addictive behaviors (Pardo et al., 2007).

#### Empirical Support in Pain Research

Within chronic pain research, the BIS–BAS model can potentially provide an important framework for understanding and explaining differences in pain related-cognitions, emotions, and behaviors (Jensen et al., 2016, 2017).

Recent studies have started reporting interesting results when examining associations among key pain-related cognitions, emotions, and behaviors and the BIS–BAS model of pain.

Pain catastrophizing, symptoms of depression and anxiety, as well as pain-related avoidance behaviors have been suggested to be BIS-related features (Day et al., 2019). A trait tendency toward BIS sensitivity has shown to be associated with pain catastrophizing (Muris et al., 2007), whereas lower BAS sensitivity has been found in fibromyalgia patients (Becerra-García and Jurado, 2014). In addition, reward responsiveness has shown to be reduced in chronic pain patients (Elvemo et al., 2015).

Emotional responses also appear to be modulated differently depending on whether the BIS or BAS system is more active. Individuals show trait differences in the activity of the two systems (De Pascalis et al., 2013), which can explain alteration in emotional regulation and in the tendency to catastrophize. BIS activity characterizes passive and fearful behavioral tendencies, including introversion, depression, anxiety, and pain (Merchan-Clavellino et al., 2019). Interestingly, a psychophysiological study (Jensen et al., 2015a) has found an association between pain catastrophizing vulnerability and frontal alpha asymmetry as a marker of BIS activity in patients with spinal cord injury and chronic pain. This provides an association between emotional regulatory disturbances and BIS–BAS activity.

Results from a recent study show that chronic pain individuals with higher BIS use suppression techniques (Serrano-Ibáñez et al., 2018). Other studies have also found associations between BIS and emotion regulation difficulties (Markarian et al., 2013; Zohreh and Ghazal, 2018).

From the BIS–BAS perspective, it has been suggested that catastrophizing could reflect the BIS-related cognitive content and process (Jensen et al., 2016). The BIS system has been hypothesized to be the neurophysiological system underlying psychological traits such as catastrophizing. Consequently, according to this hypothesis, the BIS system activates or facilitates these types of cognitive responses when facing pain (Jensen et al., 2015b, 2016).

Based on the association of BIS with behavioral inhibition and withdrawal, new studies are also supporting a relationship between pain and BIS (Jensen et al., 2017), and BIS and threat detection (Ávila and Torrubia, 2006). In a study from Jensen et al. (2015b), higher levels of pain intensity and more frequent headaches were both associated with higher BIS scores, and this association was significantly higher in individuals reporting severe headaches.

BIS and BAS systems have been suggested to be involved in the regulation of the activity in the autonomic nervous system (Beauchaine, 2001). Empirical studies have suggested that both anxiety and depression are characterized by heightened BIS activity and reduction of vagal tone (Beauchaine et al., 2007).

### Catastrophic Worry and Interoceptive Sensitivity

The fluctuating physiological state of the body can influence how individuals interpret the world (Garfinkel and Critchley, 2016). *Interoception* is defined as the sense of the physiological condition of the body, i.e., conscious awareness, emotional processes, and behavior related to afferent physiological information arising from the body (Cameron, 2001; Craig, 2003). Converging evidence are also suggesting the importance of interoception and pain (Nakamura and Chapman, 2002; Craig, 2003). *Interoceptive sensitivity* has been suggested to play a key role in the etiology and maintenance of state and trait anxiety, anxiety sensitivity, and anxiety disorders.

Patients with increased anxiety sensitivity generally report hypervigilance for somatic sensations (De Berardis et al., 2007; Olatunji et al., 2007; Anderson and Hope, 2009). A consequence of this hypervigilance is an increased self-report of somatic sensations, and a dysfunctional cognitive appraisal of these sensations with a bias toward a danger- related and catastrophizing interpretational style (Beck et al., 2005).

Negative emotions can be intensified or misinterpreted by physiological arousal, which can lead to catastrophic interpretation in anxiety and panic disorders (Teachman et al., 2010). An increased arousal observed in anxiety disorders has been observed affecting the inhibitory activity of the parasympathetic nervous system (Friedman and Thayer, 1998).

Perception of somatic sensations and subsequent catastrophizing interpretations of these sensations are particularly related to interoceptive stimuli arising from the cardiac system. Indeed, it has been proposed as a physiological mechanism underlying the link between worry and cardiovascular health. According to the *neurovisceral integration model*, vagally mediated heart rate variability (vmHRV) has been suggested as an index of emotion regulatory capacity and threat perception (Thayer and Lane, 2000). A reduction in HRV represents a breakdown of the inhibitory influences that allows for efficient self-emotional regulation.

#### Empirical Support in Pain Research

Poor emotional regulation capacities have been associated with worry and heart rate variability (HRV). Deschênes et al. (2016) showed that catastrophic worry was associated with decrease of HRV, whereas Koenig et al. (2016) found that chronic pain patients with high levels of catastrophizing were associated with low level of vagally mediated heart rate variability (vmHRV).

## Re-Composing Catastrophizing: a Proposed Integrative View

Many psychological and pain-driven theories have included catastrophizing as a central construct involved in pain and affective disturbances. We have deconstructed common elements that emerge from different theoretical models, with the aim to understand the concept of catastrophizing and its role in pain processing. Based on analysis of the literature, important key-elements emerge as fundamental to explain pain catastrophizing: emotional regulation, catastrophic worry (as repetitive negative thinking), rumination, BIS system, and interoceptive sensitivity.

Self-emotional regulatory mechanism emerges as an important process in individuals with chronic pain, due to the association with pain and negative emotions. Emotional regulation is a relative new construct in the chronic pain literature but it is important for understanding how pain patients modulate and express their emotional state, which includes which emotions an individual has, when she/he has these emotions, and how she/he experiences and expresses these emotions (Gross and Thompson, 2007). Data from a recent systematic-review (Koechlin et al., 2018) show that a small but growing number of studies are suggesting that maladaptive emotional regulation might be a risk factor for the development of chronic pain.

From the literature, we can isolate a common process, catastrophic worry (or repetitive negative thinking), which occurs when people have difficulties dealing with negative emotions. We can hypothesize that in pain patients any somatic or noxious sensations from the body will generate negative emotions, which at the same time trigger a catastrophic worry process (a *what if*… questioning style: *What if this pain doesn’t go away? What if the pain is a sign of a terrible disease? What if I will never be functional again?*). The catastrophic worry cycle is continuously reinforced through a self-focused ruminative process. The function of the catastrophic worry is to reduce negative emotions. Paradoxically, this way of processing the information increases the attention and focus on the pain that an individual wants to suppress, with the result of magnifying the problem and providing more fuel to an everlasting cycle. Furthermore, the catastrophic worry *per se* can become its own stressor to sustain the continuous cycle.

In addition, we suggest that individuals with high activity in the BIS are also the ones engaging in more catastrophic worry. Since BIS and catastrophic worry are both associated with emotional regulatory difficulties (Serrano-Ibáñez et al., 2018, 2019a), it is likely to suppose that these individuals will account differently in response to the pain experience from patients with a low BIS activity/catastrophic worry.

Since HRV has been suggested as an index of functional integrity of the neural networks implicated in the emotion-cognitive interaction (Park and Thayer, 2014) and self-regulatory capacity (Butler et al., 2006), we hypothesize that catastrophic worry individuals will have a lower HRV associated with hypervigilant and maladaptive cognitive responses to emotional stimuli, which impact on emotion regulative capacity.

Recent findings on placebo analgesia responders (De Pascalis and Scacchia, 2019) supported a negative link between HRV time-related changes and EEG-delta activity and pain reduction when reward interest was the mediator factor, suggesting that pain reduction could reflect the individual ability to self-regulate both sensorial and emotional components of pain.

## Discussion of Implications and Future Directions

The analysis of the literature has shown that a number of key-factors such as emotional regulation, catastrophic worry, rumination, BIS–BAS systems, and interoceptive sensitivity are related to catastrophizing. We have suggested an integrated view of pain catastrophizing that attempts to incorporate all these factors as interconnected processes.

Possibly, the phenomenon that we observe in certain pain patients, and we refer to as pain catastrophizing is much more complex, and it is the result of several interconnected processes and mechanisms. The analysis presented in this review shows that there is a complex interplay of psychosocial and biological mechanisms involved in pain catastrophizing.

Moving forward, research should incorporate all aforementioned key-elements possibly tested as an integrated model of catastrophizing. The one we have proposed may be likely, but other interconnections could exist, and clearly empirical and clinical research is required to test this model. From our perspective, this integrative model of catastrophizing can generate new hypotheses testing that cannot emerge from taking into consideration each single model at the time. Thus, providing a testable biopsychosocial approach.

Additionally, evidence suggests that pain catastrophizing scales cannot provide valid measures of pain catastrophizing (Crombez et al., 2020), but instead a person-centered approach would be a better way for understanding this process. By differentiating several key components, our model offers a better framework for an individualized approach. These components relate to repetitive negative thinking (Davey and Levy, 1998), catastrophic worry (Borkovec et al., 1983; Flink et al., 2013; Linton, 2013), personality characteristics related to BIS profiles (Corr, 2008; Jensen et al., 2016, 2017), and interoceptive sensitivity (Thayer and Lane, 2000; Deschênes et al., 2016; Koenig et al., 2016). However, these elements can be expanded and/or integrated into other existing models such as expectancies about future pain and disability (Peerdeman et al., 2016), attentional bias (Eccleston and Crombez, 1999; Van Damme et al., 2010) or cognitive intrusion (Attridge et al., 2015).

At present, it seems possible to hypothesize that pain catastrophizing and catastrophic worry share similarities. However, the first research question to answer is whether chronic pain patients engage in catastrophic worry processes. Exploring the worry and ruminative contents is also very important to underline the thinking style of these patients. Nonetheless, future research should directly test whether individuals with BIS-related profiles are more prone to catastrophize, and whether pain catastrophizing can be conceptualized as an emotional regulator. Viewing catastrophizing as an emotional regulatory strategy (Flink et al., 2013; Linton, 2013) will enable us to understand the cognitive-affective processes associated with the pain experience, and to develop more tailored psychological interventions that target catastrophic worry individuals, probably the ones with BIS-related profiles (Serrano-Ibáñez et al., 2019a, b).

Indeed, future clinical and experimental research should further assess the relationship between the BIS and the BAS systems and catastrophic worry. The BIS–BAS model of pain is also a relatively new model and it is mainly based on Gray’s original RST (Gray, 1982), in which the main BIS function is to regulate aversive motivation. Over the years, the theory has been revised and reconceptualized in three different components: BIS, BAS and FFFS (fight-flight-freezing-system). FFFS mediates fear-related responses to all aversive stimuli and produces avoidance and escape behaviors. BIS instead mediates anxiety-related responses and is responsible for detecting and resolving conflict between approach and avoidance (Gray and McNaughton, 2000; Corr, 2008). Jensen’s model (Jensen et al., 2016) is based on measures of BIS (Carver and White, 1994), that lacks separation between BIS-anxiety and FFFS-fear (fight-flight-freezing-system) (Corr and Cooper, 2016; De Pascalis et al., 2019). This theoretical differentiation could provide us greater insight when considering worry and anxiety as part of pain catastrophizing. Additionally, it could account for inconsistent findings when relating BIS scale to placebo and nocebo effects (Corsi and Colloca, 2017).

Finally, the BAS scale has no clear theoretical justification for its subdivision in three components, drive, reward responsiveness, and fun-seeking (Harmon-Jones et al., 2013). Therefore, further research is required to shed more light on the role of the BIS and BAS in pain.

We also suggest that further research could include psychophysiological biomarkers such as EEG/ERP and vagal mediated heart rate variability in order to increase objective assessments of biological mechanisms of pain catastrophizing. Recent research is highlighting intriguing possibilities (Jensen et al., 2015a; Deschênes et al., 2016; Koenig et al., 2016; De Pascalis and Scacchia, 2019).

Studies designed to test specific hypotheses regarding the mechanisms and processes that are behind pain catastrophizing will be extremely beneficial, since they will enable us to identify vulnerable or potential vulnerable individuals. Research that seeks to disentangle risk and vulnerability factors that contribute to pain chronicity is fundamental, as well as it is fundamental to have multidisciplinary theoretical frameworks for studying and analyzing these factors. Current pain research supports the importance of integrating psychological factors in these frameworks (Jensen and Turk, 2014).

Understanding the mechanisms and the underlying psychological dynamics will allow us to develop tailored psychological-pain related interventions that can be beneficial for specific patient characteristics. Currently, it seems probable that treatments that teach patients emotional regulation strategies could potentially reduce the negative impact of BIS activation on the ability to live with chronic pain (Serrano-Ibáñez et al., 2018). Nonetheless, future research should explore this link directly.

We believe that it is essential that we expand our knowledge in the territory of psychology and pain. Pain specialists should be able to operate using several tools that have proven useful in managing pain patients independently from their background orientation. Furthermore, these tools should be shared with all health care pain-related professionals. Regrettably, surveys are showing that although medical providers are recognizing the value of the biopsychosocial model of pain treatment, they appear to be lacking in operationalizing this model in their practice (Darnall et al., 2016, 2017). Pain psychologists could help in complementing this lack since they are more used to work within this conceptual framework. We believe that this will be highly beneficial for both clinicians and researchers in broadening up their approach to chronic pain.

## Conclusion

Pain is a health problem that requires a more interdisciplinary approach than do many other medical conditions. The experience of pain and the response to pain are the product of interrelated biological and psychosocial dynamics.

Pain catastrophizing is an important psychological factor that influences pain response and disability, but paradoxically no conceptual agreement is available at present that can explain what catastrophizing really is. From the literature, we identified several components that could clarify the construct of catastrophizing. We also presented an integrated view that can illustrate the complex interplay of biological and psychosocial factors that can shape pain vulnerability, and which can become risks factors to individuals that are prone to catastrophize.

Taken together, the present findings provide support to the view that pain catastrophizing is an emotional regulator strategy, a catastrophic worry, constituting an interplay between a ruminative process and personality trait characteristics related to the BIS system.

## Author Contributions

LP and LA-N conceptualized and wrote the manuscript. Both authors contributed to the article and approved the submitted version.

## Conflict of Interest

The authors declare that the research was conducted in the absence of any commercial or financial relationships that could be construed as a potential conflict of interest.
